# Colorectal Cancer in the U.S., 1999–2021: Declining Rates, Rising Concerns, and Persistent Disparities

**DOI:** 10.3390/diseases13120392

**Published:** 2025-12-04

**Authors:** Qais Bin Abdul Ghaffar, Sayed Maisum Mehdi Naqvi, Garrett Shields, Ebubekir Daglilar, Harleen Chela

**Affiliations:** 1 Department of Medicine, Dow International Medical College, Karachi 75280, Pakistan; 2 Mercy Hospital St. Louis, St. Louis, MO 63141, USA; 3 Division of Gastroenterology and Hepatology, Charleston Area Medical Center, West Virginia University, Charleston, WV 25304, USA

**Keywords:** colorectal neoplasms, incidence, mortality, mortality-to-incidence ratio, health disparities, early-onset colorectal cancer, joinpoint regression, SEER program

## Abstract

**Background:** Colorectal cancer (CRC) incidence and mortality have declined in the United States over the past two decades, yet disparities persist by age, sex, race/ethnicity, and geography. To characterize population-level survival signals, we examined trends in age-adjusted incidence rates (AAIR), mortality rates (AAMR), and the mortality-to-incidence ratio (AAMIR) from 1999 to 2021, stratified by key subgroups. **Methods:** This retrospective analysis utilized de-identified data from the CDC WONDER United States Cancer Statistics database, encompassing incident CRC cases (SEER codes 21041–21052) and deaths (ICD-10 codes C18–C20) in adults aged 20 years and older. Age-adjusted rates (per 100,000, 2000 U.S. standard population) and AAMIR were calculated using Stata 17.0. Joinpoint regression identified trends (annual or average annual percent change [APC/AAPC], *p* < 0.05). **Results:** Among 3,489,881 cases and 1,225,986 deaths, AAIR decreased from 78.24 (1999) to 50.79 (2021; AAPC: −2.20%, 95% CI: −2.52 to −1.89), AAMR decreased from 29.34 to 17.92 (AAPC: −2.33%, −2.46 to −2.20), and AAMIR from 0.375 to 0.353 (AAPC: −0.08%, −0.47 to 0.30; *p* = 0.669). Women showed a significant AAMIR decline (AAPC: −0.29%), unlike men (AAPC: 0.07%). Young adults (20–39 years) had rising AAIR (AAPC: 2.42%) and AAMR (0.87%) but improving AAMIR (AAPC: −1.71%). Non-Hispanic Black individuals had the highest AAMIR (0.400 in 2021; AAPC: −0.54%). The Northeast had the most favorable AAMIR trend (AAPC: −0.40%), while the Midwest, South, and West were stable. States like New Jersey and Massachusetts achieved low AAMIR (0.292 and 0.304 in 2021), contrasting with Nebraska and Arizona (0.402 in both). **Conclusions:** Although colorectal cancer incidence and mortality have declined substantially in the United States from 1999 to 2021, the mortality-to-incidence ratio improved only marginally and remained markedly uneven across subgroups. Targeted interventions—enhancing screening and treatment access for men, racial/ethnic minorities, younger adults, and high-burden regions and states—can promote equitable outcomes.

## 1. Introduction

Colorectal cancer (CRC) remains a major public health concern in the United States. It is common, deadly, and, despite real progress, continues to rank among the leading causes of cancer death [1]. Over the past two decades, incidence and mortality have generally fallen as prevention, as early detection and treatment have improved [1,2]. Yet the headline gains can blur important differences. Risk is shifting toward younger adults, and the benefits of timely diagnosis and high-quality therapy have not reached all communities equally, patterns that are especially visible when urban and rural areas are compared [1,2,3]. Although numerous national reports describe CRC incidence and mortality trends by demographic and geographic subgroup [1,2,4,5,6,7], they rarely evaluate how mortality tracks incidence within these groups over time using metrics such as the mortality-to-incidence ratio, so population-level mortality-to-incidence patterns remain under-characterized.

Screening helps explain much of the progress. Stool tests and colonoscopy prevent cancer by removing adenomas and moving diagnoses to earlier stages, where outcomes are better [2,8]. In 2021, responding to rising risk under age 50, the US Preventive Services Task Force lowered the average-risk screening age from 50 to 45 to curb early-onset disease [8,9]. Treatment advances also matter. Modern multimodality care and biomarker-directed options, most notably immune checkpoint blockade for MSI-H/dMMR tumors, have improved outcomes for selected patients [10]. Even so, early-onset CRC continues to rise, and geographic and sociodemographic disparities remain stubborn [1,2,3,9].

To see where progress is, and is not, translating into population benefit, it is necessary to examine not only incidence and mortality separately but also how they balance within groups over time. Prior work has used the mortality-to-incidence ratio and its age-adjusted analog, the age-adjusted mortality-to-incidence ratio (AAMIR), defined as the age-adjusted mortality rate (AAMR) divided by the age-adjusted incidence rate (AAIR), with both rates standardized to the 2000 U.S. standard population, as pragmatic, population-level indicators of cancer-control performance when survival data are incomplete or delayed [11,12]. By summarizing how far mortality has fallen relative to incidence within a given population, AAMIR can distinguish groups in which similar incidence rates are accompanied by very different mortality experiences, or in which substantial incidence reductions have not been matched by proportional mortality declines, thereby highlighting potential “translation gaps” along the prevention–diagnosis–treatment continuum [11,12]. At the same time, methodological work has shown that AAMIR is not a valid substitute for patient-level survival; it is influenced by changes in incidence, stage mix, and data quality and must be interpreted alongside conventional incidence, mortality, and survival measures [13]. Building on these national descriptions of CRC incidence and mortality by demographic and geographic subgroup [1,2,4,5,6,7], we note that corresponding AAMIR patterns across sex, age, race/ethnicity, region, and state are less well characterized. By jointly examining age-adjusted incidence (AAIR), mortality (AAMR), and AAMIR across these subgroups from 1999 to 2021, we aim to describe how the mortality-to-incidence balance has evolved in the United States, identify populations and jurisdictions where mortality remains disproportionately high relative to incidence, and highlight targets for more equitable prevention, early detection, and treatment implementation.

## 2. Methods

### 2.1. Study Design

This retrospective study analyzed incidence and mortality data for colorectal cancer (CRC) from the Centers for Disease Control and Prevention’s Wide-Ranging Online Data for Epidemiologic Research (CDC WONDER) platform, specifically the United States Cancer Statistics (USCS) database, spanning 1999 to 2021 [14]. The USCS database integrates population-based cancer registry data from the National Program of Cancer Registries (NPCR) and the Surveillance, Epidemiology, and End Results (SEER) Program, providing near-complete coverage of cancer incidence and mortality across the United States.

Population coverage increased from approximately 78% in 1999 to 100% by 2005 and has remained ≥99% thereafter. All rates presented are based on registries that met USCS publication criteria for each year (≥90% case ascertainment, ≤5% of cases reported by death certificate only, etc.). State-level estimates for early years (1999–2004) reflect only the participating registries for those years; however, because national and regional trends are dominated by large, continuously participating states and because coverage reached near-universal levels by 2005, temporal comparisons are considered robust and are used in official CDC and American Cancer Society reports. The study included incident cases among adults aged ≥20 years diagnosed with CRC, identified using SEER site and histology recode codes 21041–21052, which correspond to malignant neoplasms of the colon and rectum [15]. The analysis included all malignant colorectal cancers (colon: C18.0–C18.9, C26.0; rectum: C19.9, C20.9) reported to registries participating in the United States Cancer Statistics (USCS) database. Cases were identified using the International Classification of Diseases for Oncology, third edition (ICD-O-3) site, histology recode 21041–21052, which corresponds to invasive (malignant) behavior only. In situ cases, benign tumors, and tumors of uncertain malignant potential were excluded by the definition of these codes. Only first primary colorectal tumors (sequence number 00 or 01) were counted as one primary per person; subsequent primaries in the same individual were excluded. Cases diagnosed by death certificate only or autopsy only were excluded by the USCS publication criteria.

For mortality, deaths were identified using the International Classification of Diseases, 10th revision (ICD-10) codes C18–C20 (malignant neoplasms of colon, rectosigmoid junction, and rectum) as the underlying cause of death [16]. As the data were de-identified and publicly available, Institutional Review Board (IRB) approval was not required. The study adhered to the Strengthening the Reporting of Observational Studies in Epidemiology (STROBE) guidelines [17]. Data were accessed in September 2025 from the CDC WONDER USCS database. All data were publicly available and fully de-identified; no author had access to identifiable private information at any stage of the research.

### 2.2. Data Abstraction

The original data presented in the study are openly available in the CDC WONDER USCS database [14]. Extracted variables included year of diagnosis or death, age at diagnosis or death (stratified as 20–39, 40–64, and ≥65 years), gender (male, female), race/ethnicity, geographic region, and state. Race/ethnicity was classified using the standard categories provided by the CDC WONDER United States Cancer Statistics database, which follows Office of Management and Budget (OMB) standards and bridges multiple-race data to single-race categories when necessary. The categories used were: Non-Hispanic White (single race), Non-Hispanic Black (single race), Non-Hispanic American Indian/Alaska Native (single race), Non-Hispanic Asian or Pacific Islander (includes Asian and Native Hawaiian or Other Pacific Islander, single race), and Hispanic (all races). Individuals reporting more than one race are allocated to a single race using the National Center for Health Statistics bridging algorithm as implemented in CDC WONDER; no additional re-coding or custom grouping was performed by the authors. Data for the “Non-Hispanic Multiple Race” and “Non-Hispanic Unknown Race” categories are included in the overall totals but were not presented separately because of small numbers and unstable rates in many years.

Geographic regions were defined according to the U.S. Census Bureau’s four-region classification (Northeast, Midwest, South, West) [18]. Urban–rural classification was not included in this analysis, but could be incorporated using the 2013 National Center for Health Statistics (NCHS) Urban–Rural Classification Scheme if required [19].

### 2.3. Statistical Analysis

Age-adjusted rates (AAIR and AAMR) and their standard errors were calculated in CDC WONDER using the direct method and the 2000 U.S. standard population [20]. Annual age-adjusted mortality-to-incidence ratios (AAMIR) were computed as AAMR ÷ AAIR. Joinpoint regression used weighted least squares with weights equal to the inverse of the variance of the age-adjusted rates (i.e., the standard weighted procedure in the Joinpoint software); confidence intervals for APC and AAPC were calculated using the normal approximation on the log-transformed rates, as implemented by default in Joinpoint version 5.0.2 [21]. Trends in age-adjusted incidence rates (AAIR), mortality rates (AAMR), and mortality-to-incidence ratios (AAMIR) were analyzed using the Joinpoint Regression Program version 5.0.2 (National Cancer Institute, Bethesda, MD, USA). A maximum of three joinpoints (four line segments) was allowed for all stratified analyses (sex, age group, race/ethnicity, region, and state); a maximum of five joinpoints was permitted for the overall national series. Model selection was performed using the permutation test with 4499 permutations and an overall significance level of 0.05. No additional correction for multiple comparisons was applied, consistent with standard practice in NCI cancer-trend analyses. Weighted least-squares estimation was used, with weights equal to the inverse variance of the age-adjusted rates. Confidence intervals for annual percent change (APC) and average annual percent change (AAPC) were calculated using the normal approximation on the log-transformed rates. The number of joinpoints identified, their locations (years), segment-specific annual percent changes (APC), and average annual percent changes (AAPC) with 95% confidence intervals for AAIR, AAMR, and AAMIR are provided in Supplemental Tables S1 (AAMIR), S2 (AAIR), and S3 (AAMR).

### 2.4. Rationale for Selected Parameters and Stratifications

We examined age-adjusted incidence rates (AAIR) to capture prevention and early-detection progress, age-adjusted mortality rates (AAMR) to reflect overall survival gains, and the age-adjusted mortality-to-incidence ratio (AAMIR) as a pragmatic population-level proxy for how effectively incident cases are translated into survival when individual-level data are delayed. Stratification by sex, age group, race/ethnicity, Census region, and state was performed because these are the major, well-documented axes of colorectal cancer disparity in the United States.

## 3. Results

From 1999 to 2021, colon and rectum cancer accounted for 3,489,881 cases (51.8% male, 48.2% female) and 1,225,986 deaths (51.6% male, 48.4% female) (Supplemental Table S4). The age-adjusted incidence rate (AAIR) decreased from 78.24 in 1999 to 50.79 in 2021 (AAPC: −2.20% [−2.52, −1.89], *p* < 0.001), and the age-adjusted mortality rate (AAMR) declined from 29.34 in 1999 to 17.92 in 2021 (AAPC: −2.33% [−2.46, −2.20], *p* < 0.001). The age-adjusted mortality-to-incidence ratio (AAMIR) slightly decreased from 0.375 in 1999 to 0.353 in 2021 (AAPC: −0.08% [−0.47, 0.30], *p* = 0.669) (Table 1; Supplemental Table S4).

### 3.1. Gender-Specific Trends

**Figure 1:**
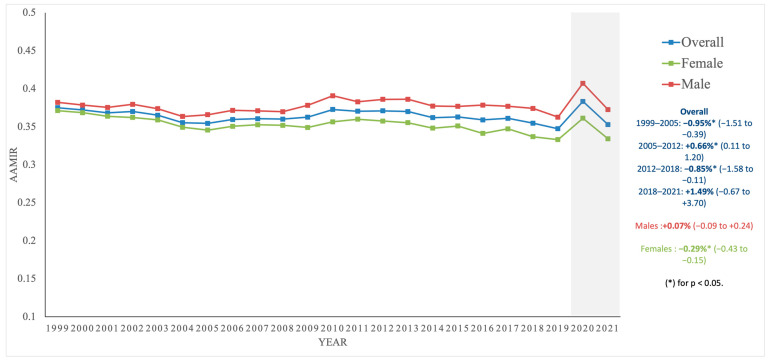
Trends in age-adjusted mortality-to-incidence ratios (AAMIR) for colorectal cancer overall and by sex, United States, 1999–2021. The figure displays annual AAMIR values over time, with separate lines for the overall population, males, and females. The light gray shaded area indicates the COVID-19 pandemic period (2020–2021).

From 1999 to 2021, males had higher incidence and mortality rates than females (Figure 1). For males, AAIR fell from 93.48 in 1999 to 57.32 in 2021 (AAPC: −2.44% [−2.76, −2.13], *p* < 0.001), AAMR decreased from 35.72 in 1999 to 21.36 in 2021 (AAPC: −2.36% [−2.65, −2.08], *p* < 0.001), and AAMIR remained stable from 0.382 in 1999 to 0.373 in 2021 (AAPC: 0.07% [−0.09, 0.24], *p* = 0.353). For females, AAIR declined from 67.04 in 1999 to 45.02 in 2021 (AAPC: −2.06% [−2.38, −1.74], *p* < 0.001), AAMR dropped from 24.89 in 1999 to 15.04 in 2021 (AAPC: −2.40% [−2.55, −2.26], *p* < 0.001), and AAMIR decreased from 0.371 in 1999 to 0.334 in 2021 (AAPC: −0.29% [−0.43, −0.15], *p* < 0.001) (Table 1; Supplemental Table S5).

### 3.2. Age Group Trends

**Figure 2:**
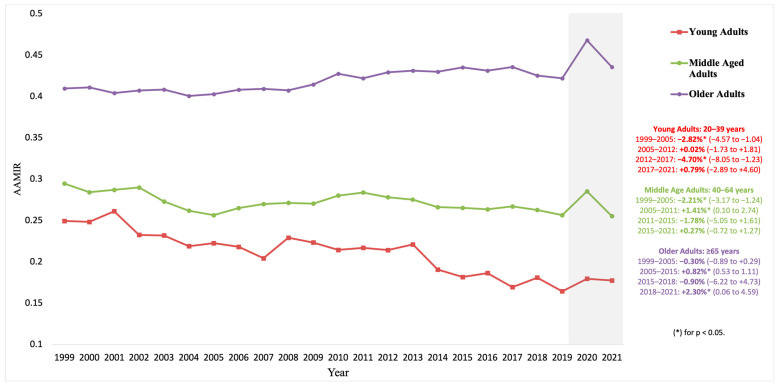
Trends in age-adjusted mortality-to-incidence ratios (AAMIR) for colorectal cancer by age group, United States, 1999–2021. The figure shows annual AAMIR trends segmented by age: 20–39 years, 40–64 years, and ≥65 years. Joinpoint regression lines indicate significant changes in trends. The light gray shaded area indicates the COVID-19 pandemic period (2020–2021).

From 1999 to 2021, among young adults (20–39 years), AAIR increased from 3.68 in 1999 to 6.45 in 2021 (AAPC: 2.42% [1.76, 3.08], *p* < 0.001), with a sharp rise from 2013 to 2016 (APC: 6.45% [1.55, 11.60], *p* = 0.013), AAMR slightly increased from 0.92 in 1999 to 1.14 in 2021 (AAPC: 0.87% [0.63, 1.11], *p* < 0.001), and AAMIR decreased from 0.249 in 1999 to 0.177 in 2021 (AAPC: −1.71% [−2.85, −0.56], *p* = 0.004). For middle-aged adults (40–64 years), AAIR slightly decreased from 52.63 in 1999 to 51.15 in 2021 (AAPC: −0.39% [−0.72, −0.05], *p* = 0.024), AAMR declined from 15.51 in 1999 to 13.06 in 2021 (AAPC: −0.82% [−1.28, −0.36], *p* = 0.001), and AAMIR remained stable from 0.295 in 1999 to 0.255 in 2021 (AAPC: −0.48% [−1.20, 0.25], *p* = 0.196). For older adults (65+ years), AAIR significantly decreased from 308.13 in 1999 to 154.22 in 2021 (AAPC: −3.44% [−3.61, −3.27], *p* < 0.001), AAMR dropped from 126.23 in 1999 to 67.17 in 2021 (AAPC: −2.98% [−3.12, −2.85], *p* < 0.001), and AAMIR slightly increased from 0.410 in 1999 to 0.436 in 2021 (AAPC: 0.48% [−0.28, 1.24], *p* = 0.215) (Figure 2; Table 1; Supplemental Table S6).

### 3.3. Racial and Ethnic Group Trends

**Figure 3:**
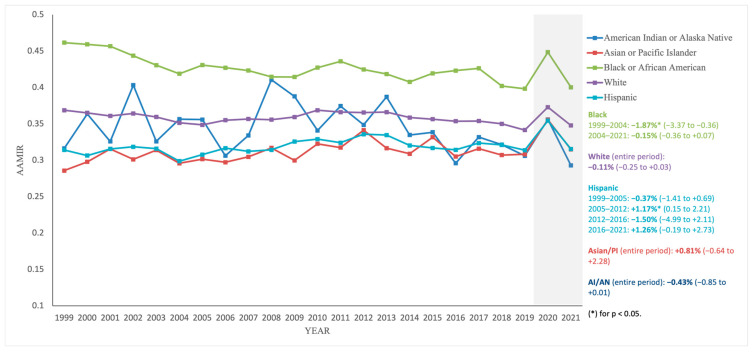
Trends in age-adjusted mortality-to-incidence ratios (AAMIR) for colorectal cancer by race/ethnicity, United States, 1999–2021. The figure illustrates annual AAMIR values by racial/ethnic group: Non-Hispanic White, Non-Hispanic Black, Hispanic, Non-Hispanic Asian/Pacific Islander, and Non-Hispanic American Indian/Alaska Native. The light gray shaded area indicates the COVID-19 pandemic period (2020–2021).

From 1999 to 2021, Non-Hispanic Black individuals had the highest AAMIR, despite a decrease from 0.462 in 1999 to 0.400 in 2021 (AAPC: −0.54% [−0.90, −0.18], *p* = 0.003), with AAIR falling from 87.53 in 1999 to 57.88 in 2021 (AAPC: −2.05% [−2.50, −1.61], *p* < 0.001) and AAMR dropping from 40.40 in 1999 to 23.16 in 2021 (AAPC: −2.67% [−2.77, −2.57], *p* < 0.001). For Non-Hispanic White individuals, AAIR decreased from 78.68 in 1999 to 51.66 in 2021 (AAPC: −2.11% [−2.40, −1.82], *p* < 0.001), and AAMR dropped from 29.01 in 1999 to 17.97 in 2021 (AAPC: −2.22% [−2.50, −1.93], *p* < 0.001). The Hispanic population had AAIR fall from 64.54 in 1999 to 47.47 in 2021 (AAPC: −1.73% [−1.96, −1.50], *p* < 0.001), AAMR decrease from 20.28 in 1999 to 14.97 in 2021 (AAPC: −1.51% [−1.62, −1.40], *p* < 0.001), and AAMIR remain stable from 0.314 in 1999 to 0.315 in 2021 (AAPC: 0.28% [−0.48, 1.05], *p* = 0.472). Non-Hispanic Asian or Pacific Islander individuals saw AAIR decline from 59.44 in 1999 to 41.55 in 2021 (AAPC: −2.07% [−2.31, −1.82], *p* < 0.001), AAMR drop from 16.99 in 1999 to 13.08 in 2021 (AAPC: −1.73% [−1.95, −1.50], *p* < 0.001), and AAMIR remain stable from 0.286 in 1999 to 0.315 in 2021 (AAPC: 0.81% [−0.64, 2.28], *p* = 0.276). Non-Hispanic American Indian or Alaska Native individuals had a slight AAIR decrease from 67.54 in 1999 to 62.11 in 2021 (AAPC: −0.27% [−0.58, 0.04], *p* = 0.083), AAMR declined from 21.36 in 1999 to 18.19 in 2021 (AAPC: −0.84% [−1.36, −0.32], *p* = 0.003), and AAMIR showed a non-significant decline from 0.316 in 1999 to 0.293 in 2021 (AAPC: −0.43% [−0.85, 0.01], *p* = 0.053) (Figure 3; Table 1; Supplemental Table S7).

### 3.4. Regional Trends

**Figure 4:**
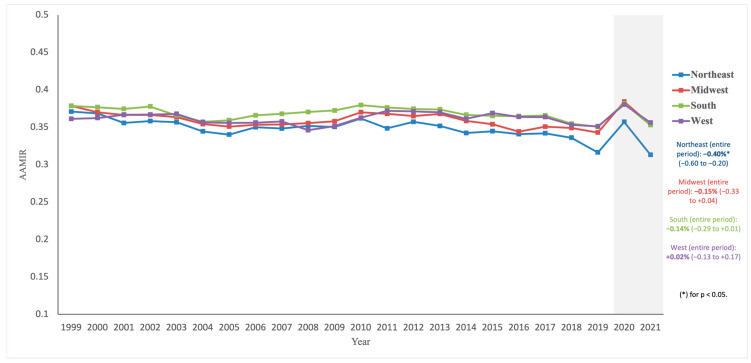
Trends in age-adjusted mortality-to-incidence ratios (AAMIR) for colorectal cancer by geographic region, United States, 1999–2021. The figure depicts annual AAMIR trends by U.S. region: Northeast, Midwest, South, and West. The light gray shaded area indicates the COVID-19 pandemic period (2020–2021).

From 1999 to 2021, the Northeast had the most favorable AAMIR trend, decreasing from 0.371 in 1999 to 0.313 in 2021 (AAPC: −0.40% [−0.60, −0.20], *p* < 0.001), with AAIR falling from 86.56 in 1999 to 50.62 in 2021 (AAPC: −2.73% [−3.05, −2.42], *p* < 0.001) and AAMR dropping from 32.10 in 1999 to 15.86 in 2021 (AAPC: −3.21% [−3.38, −3.04], *p* < 0.001). The Midwest, South, and West showed stable AAMIR trends (AAPC: −0.15% [−0.33, 0.04], *p* = 0.111; AAPC: −0.14% [−0.29, 0.01], *p* = 0.062; AAPC: 0.02% [−0.13, 0.17], *p* = 0.767, respectively), with AAIR declines from 82.21 to 53.34, 75.64 to 53.72, and 70.67 to 47.63, and AAMR declines from 31.12 to 18.86, 28.62 to 18.96, and 25.52 to 16.96 (all *p* < 0.001) (Figure 4; Table 1; Supplemental Table S8).

### 3.5. State-Specific Trends

From 1999 to 2021, states with the highest age-adjusted mortality-to-incidence ratio (AAMIR) values (Supplemental Figure S1), indicating less favorable trends, were Nebraska (0.364 in 1999 to 0.402 in 2021, AAPC: 0.51% [−1.22, 2.27], *p* = 0.564, with a significant rise from 2016 to 2021, APC: 5.06% [2.28, 7.93], *p* = 0.002), Vermont (0.367 in 1999 to 0.352 in 2021, AAPC: 0.40% [−0.36, 1.16], *p* = 0.286), Oklahoma (0.387 in 1999 to 0.371 in 2021, AAPC: 0.37% [0.02, 0.73], *p* = 0.040), Arizona (0.375 in 1999 to 0.402 in 2021, AAPC: 0.28% [0.02, 0.54], *p* = 0.035), and Rhode Island (0.347 in 1999 to 0.324 in 2021, AAPC: 0.27% [−0.24, 0.78], *p* = 0.288). Their AAIRs and AAMRs declined significantly (Nebraska: 85.78 to 52.95, AAPC: −2.33% [−3.86, −0.78]; 31.23 to 21.26, AAPC: −1.75% [−2.44, −1.06]; Vermont: 83.93 to 46.75, AAPC: −2.87% [−3.25, −2.50]; 30.79 to 16.44, AAPC: −2.47% [−3.04, −1.89]; Oklahoma: 75.63 to 60.75, AAPC: −1.53% [−1.81, −1.25]; 29.28 to 22.55, AAPC: −1.18% [−1.46, −0.90]; Arizona: 65.54 to 42.95, AAPC: −2.02% [−2.24, −1.80]; 24.57 to 17.27, AAPC: −1.80% [−2.24, −1.35]; Rhode Island: 94.31 to 44.47, AAPC: −3.36% [−4.09, −2.62]; 32.75 to 14.43, AAPC: −3.46% [−3.84, −3.08]; all *p* < 0.001). States with the lowest AAMIR values, reflecting more favorable trends, were New Jersey (0.369 in 1999 to 0.292 in 2021, AAPC: −0.64% [−0.93, −0.35], *p* < 0.001), New Hampshire (0.423 in 1999 to 0.319 in 2021, AAPC: −0.63% [−1.19, −0.05], *p* = 0.034), Massachusetts (0.386 in 1999 to 0.304 in 2021, AAPC: −0.50% [−0.79, −0.22], *p* = 0.002), Maryland (0.403 in 1999 to 0.369 in 2021, AAPC: −0.44% [−0.70, −0.17], *p* = 0.002), and Minnesota (0.359 in 1999 to 0.306 in 2021, AAPC: −0.43% [−0.75, −0.11], *p* = 0.011). Their AAIRs and AAMRs also declined significantly (New Jersey: 89.12 to 55.72, AAPC: −2.55% [−2.95, −2.14]; 32.87 to 16.27, AAPC: −3.28% [−3.44, −3.11]; New Hampshire: 76.70 to 46.63, AAPC: −2.69% [−3.13, −2.24]; 32.46 to 14.87, AAPC: −3.32% [−3.79, −2.85]; Massachusetts: 81.80 to 45.51, AAPC: −2.85% [−3.51, −2.18]; 31.58 to 13.86, AAPC: −3.70% [−4.08, −3.33]; Maryland: 78.01 to 49.23, AAPC: −2.31% [−2.80, −1.81]; 31.46 to 18.14, AAPC: −2.80% [−3.17, −2.42]; Minnesota: 73.37 to 52.52, AAPC: −1.98% [−2.23, −1.73]; 26.32 to 16.05, AAPC: −2.44% [−2.73, −2.14]; all *p* < 0.001) (Supplemental Table S9).

## 4. Discussion

Between 1999 and 2021, U.S. colorectal cancer (CRC) indicators shifted meaningfully. Age-adjusted incidence and mortality declined nationwide [1,2,8], whereas the age-adjusted mortality-to-incidence ratio (AAMIR), which summarizes how mortality tracks incidence at the population level [11,12], changed little overall, with a small, statistically nonsignificant decrease. This pattern indicates numerical near-stability in the mortality-to-incidence balance rather than a clearly demonstrable improvement in survival. AAMIR is sensitive to changes in case mix, stage at diagnosis, and data completeness and should therefore be interpreted alongside its components rather than as a proxy for individual-level survival [11,13]. Within that constraint, our findings suggest that prevention and early detection gains may have progressed faster than improvements in outcomes after diagnosis [1,2,8]. Likely contributors include case-mix differences among unscreened groups, comorbidity burdens that limit treatment intensity, and uneven access to guideline-concordant therapy [1,2]. Period effects that transiently elevate mortality independent of incidence, such as COVID-19–related disruptions in screening and cancer care during 2020–2021, may also have contributed to the numerical near-stability in AAMIR [2,22]. Published SEER-based reports similarly document gradual improvements in CRC survival with persistent racial and geographic disparities [1,2], which is directionally consistent with declining mortality and the limited AAMIR change we observe, although our analysis did not directly examine patient-level survival. Overall, these trends point to the need for strategies that extend beyond screening alone to reliably improve outcomes after diagnosis.

Sex-based differences persisted throughout the study period [1,2]. Women experienced a more favorable AAMIR trajectory than men, reflecting a comparatively larger decline in mortality relative to incidence, whereas men showed little change in this ratio despite declining rates in both components. Prior work suggests that, compared with women, men have lower CRC screening uptake, higher burdens of lifestyle-related risk factors and metabolic comorbidities, and less favorable stage distribution at diagnosis [1,23,24,25]. Large comparative analyses also indicate sex-specific patterns in tumor localization: men more often present with distal and rectal tumors, whereas women are more likely to have proximal/right-sided cancers, which tend to display more aggressive biological features and worse prognosis [24,26]. In contrast, women show higher adherence to screening programs [23,25] and may engage earlier with healthcare systems, facilitating earlier diagnosis and more complete receipt of multimodality therapy [24,25]. Biological sex differences involving sex hormones and the gut microbiome could further modulate risk and outcomes [27], but cannot be evaluated directly in our data. Taken together, differences in screening compliance, tumor localization, comorbidity burden, and underlying biology provide plausible explanations for the more favorable AAMIR trajectory observed in women compared with men, although these interpretations remain hypothesis-generating given the ecologic design and lack of individual-level screening and treatment information.

Racial and ethnic gradients were also evident. Non-Hispanic Black populations carried the highest AAMIR, despite substantial declines in both incidence and mortality, reflecting persistent inequities in how effectively reductions in disease occurrence translate into mortality gains [1,2,4]. Contributing factors include lower colonoscopy uptake, socioeconomic barriers, higher prevalence of comorbidities such as diabetes and hypertension, and reduced access to comprehensive oncology services [2,4,5,23]. Hispanic and American Indian/Alaska Native groups had flat or borderline AAMIR changes despite falling AAIR and AAMR, suggesting that access challenges and underinsurance continue to blunt improvements in the mortality-to-incidence balance [2,23,28,29]. Non-Hispanic Asian/Pacific Islanders showed a non-significant upward drift in AAMIR despite declines in both AAIR and AAMR, which may reflect differences in tumor biology, acculturation-related risk factors, or disparities in treatment access [4]. Addressing these inequities will require culturally tailored outreach, expansion of insurance coverage, and enhanced investment in community-based CRC prevention and treatment programs, including patient navigation to improve diagnostic follow-up after abnormal screening [30,31].

Age-stratified results revealed distinct dynamics. Among younger adults (20–39 years), incidence rose, most notably around 2013–2016, yet mortality increased more slowly than incidence, implying an improving AAMIR and a more favorable mortality-to-incidence balance despite rising burden [1,2]. This divergence between rising incidence and more slowly rising mortality may be consistent with better recognition and management of early-onset CRC, but also with broader etiologic shifts. Recent syntheses by Siegel and colleagues and others highlight earlier and more sustained exposure to obesogenic environments, physical inactivity, Westernized dietary patterns, antibiotic-associated alterations of the gut microbiome, and inherited susceptibility as leading hypotheses for the rising burden in younger adults [1,9]. Within midlife adults, patterns around ages 45–54 years are particularly salient: in this group, recent increases in incidence without proportional rises in mortality may partly reflect earlier detection following the 2021 USPSTF decision to lower the average-risk screening age from 50 to 45 [8,9], although these trends are recent and may also reflect underlying burden, greater diagnostic awareness, or reporting variability rather than definitive screening effects. Overall, adults aged 40–64 years experienced parallel declines in incidence and mortality with largely stable AAMIR, consistent with incremental improvements moderated by comorbidity and varied treatment adherence [1,2]. In older adults (≥65 years), large declines in incidence and mortality, consistent with reports that declines among older adults have recently slowed [1,2,32], were accompanied in our data by little recent change in AAMIR, suggesting limited additional improvement in the mortality-to-incidence balance in the most recent period. These findings underscore the need for age-tailored strategies, including expedited diagnosis and follow-up for younger adults and geriatric-informed treatment pathways for older adults [32,33], in whom AAMIR appears to have plateaued.

Geography added another layer of variation. The Northeast was the only region with a significantly favorable AAMIR trajectory, whereas the Midwest, South, and West remained statistically flat despite pronounced declines in both incidence and mortality [2,5,6,7]. This indicates that, in most regions, mortality has not fallen faster than incidence to a degree that materially improves the mortality-to-incidence balance. Regional disparities likely reflect differences in screening uptake (with several Northeastern states achieving some of the highest up-to-date CRC screening coverage nationally), Medicaid expansion and insurance coverage, patterns of risk factors, healthcare infrastructure, and the availability of specialized oncology centers [2,5,6,7,34,35,36]. The Northeast’s advantage may stem from earlier adoption of organized screening, higher colonoscopy use, and greater access to tertiary cancer centers [2,5,7,34], whereas less favorable patterns elsewhere may be tied to socioeconomic disadvantage, rural access gaps, and differences in state-level health policy implementation [5,6,7,36]. These findings underscore the importance of region-specific strategies to reduce inequities.

State-level patterns sharpened these contrasts [2,5,6]. New Jersey, New Hampshire, Massachusetts, Maryland, and Minnesota combined favorable AAMIR trajectories (with low recent values) and continued reductions in incidence and mortality, marking them as leaders in translating disease-occurrence declines into mortality reductions. In contrast, Nebraska, Oklahoma, and Arizona exhibited unfavorable AAMIR trajectories, most notably a post-2016 surge in Nebraska, despite significant declines in AAIR and AAMR. This pattern suggests gaps along the screening-to-treatment continuum, from completion of diagnostic colonoscopy after abnormal tests to timely initiation and completion of therapy, rather than a lack of progress in incidence alone. Contextual state reports underscore these concerns as Oklahoma continues to rank among the highest in CRC mortality [37], and Arizona reports recent declines from a comparatively high burden with ongoing needs in early-stage detection and follow-up [38], while Nebraska county-level death-rate profiles highlight persistent within-state variation [39]. Variability in Medicaid expansion, colonoscopy screening rates, and the distribution of tertiary oncology centers likely contributes to these differences [2,5,6,34,36]. Together, these findings point to a need for state-specific interventions that explicitly account for structural differences in access and delivery [5,6].

Therefore, our results point to several concrete, data-driven priorities. Sex gaps could be narrowed by improving male engagement in screening and treatment completion [2,23,24,25,26,27]. Racial and ethnic inequities, especially the persistently highest AAMIR among Non-Hispanic Black populations, call for sustained investment in patient navigation, rapid diagnostic follow-up after abnormal stool tests or colonoscopy, and equitable access to guideline-concordant therapy [1,2,4,5,30,31,32]. Age-tailored strategies are needed to expedite diagnosis in younger adults, whose incidence is rising, while implementing geriatric-oncology pathways for adults ≥ 65 years, in whom AAMIR gains have plateaued despite large absolute declines in incidence and mortality [1,2,9,32,33]. Regionally, replicating the Northeast’s combination of higher screening uptake and access to tertiary centers, while expanding timely colonoscopy and multidisciplinary care in the Midwest, South, and West, may help close geographic gaps [2,5,6,7,34,36]. At the state level, intensified efforts are warranted in Nebraska, Oklahoma, and Arizona, where AAMIR trends are unfavorable, while sustaining and learning from the favorable patterns in New Jersey, New Hampshire, Massachusetts, Maryland, and Minnesota [5,6,37,38,39]. Because AAMIR reflects the interplay between incidence and mortality at the population level rather than survival per se [11,12,13], coordinated investment across the screening, diagnostic, and treatment continuum in these priority populations and geographies offers a rational path to improving both components and, ultimately, CRC outcomes [1,2,11,12,31].

Because this analysis is ecological and relies on subgroup-level rates rather than individual patient data, the patterns we report may obscure substantial heterogeneity in stage at diagnosis, comorbidities, and treatment within each demographic and geographic category. Favorable AAMIR values at the regional or state level could still coexist with persistent survival gaps along lines of insurance coverage, neighborhood deprivation, or rurality, and associations observed at the group level may not apply to individual patients (ecological fallacy). Our findings should therefore be interpreted as contextual indicators of cancer-control performance rather than direct evidence of individual-level risk or treatment effects. Future studies that link population-based registries with claims and electronic health records will be essential to test mechanistic hypotheses regarding screening uptake, diagnostic delay, and treatment receipts that are only indirectly captured by AAMIR, and to refine the targets we identify for strengthening cancer-control systems.

### Limitations

This study has several limitations. First, incidence and mortality data were sourced from separate national registries, and AAMIR was calculated by combining these aggregate, age-adjusted rates. Because these registries differ in coverage, data completeness, and reporting timelines, especially in small-population or under-resourced states, some degree of underreporting, including racial and ethnic misclassification, and denominator misalignment may have introduced instability into subgroup and state-level estimates, particularly where case counts were small. Temporal changes in registry coverage and completeness over the study period may also have influenced estimates, especially in earlier years, so small absolute differences between states or subgroups should be interpreted cautiously.

Second, although AAMIR is useful for summarizing how mortality relates to incidence at the population level, it is not a measure of survival and cannot distinguish improved survival from earlier diagnosis, stage migration, changes in case mix, differences in receipt of treatment, or variation in data quality. As such, it should be interpreted as a population-level contextual metric rather than a proxy for individual-level survival. Because CDC WONDER bridges multiracial individuals to a single race using an established algorithm, our analysis could not separately examine trends among persons identifying with more than one race.

Third, this was an ecological analysis using registry-based aggregate data without access to individual-level information on tumor stage, anatomic subsite (colon vs. rectum), molecular characteristics, comorbidities, treatment patterns, health insurance, socioeconomic status, or urban–rural residence. Therefore, we could not adjust for potential confounders, investigate underlying mechanisms, or infer causality, and the potential for ecological fallacy remains. Fourth, the age groupings used (20–39, 40–65, and ≥65 years) were selected to balance interpretability with statistical stability but may mask meaningful variation within narrower, policy-relevant bands, including adults aged 45–54 years who recently became eligible for average-risk screening. Fifth, because colon and rectal cancers were analyzed together, we were unable to explore subsite-specific differences.

Finally, disruptions related to the COVID-19 pandemic in 2020–2021 (visually marked by the light-gray shaded area in Figure 1, Figure 2, Figure 3 and Figure 4) represent only 2 of 23 years studied (8.7%) and are unlikely to materially alter the reported long-term trends, consistent with recent CDC and American Cancer Society surveillance reports that retain these years. Short follow-up for the most recent calendar years further limits interpretation of tail trends, and recent AAPC estimates should be interpreted with caution. Future work linking registry and clinical datasets, incorporating stage, treatment, and survival, and examining disparities across more granular geographic, age, and tumor-specific strata will be important to extend and refine these findings.

## 5. Conclusions

From 1999 to 2021, U.S. colorectal cancer incidence and mortality declined overall, but the mortality-to-incidence balance, summarized by AAMIR, changed little and remained uneven across populations. Women, younger adults, and residents of several Northeastern states experienced more favorable combinations of declining incidence, mortality, and AAMIR, whereas men, older adults, Non-Hispanic Black and American Indian/Alaska Native populations, and many Midwestern and Southern states had persistently higher or flat AAMIR levels. 

These patterns indicate that reducing incidence alone is insufficient. Improving population-level outcomes will require targeted efforts to increase screening uptake and diagnostic follow-up, ensure timely, guideline-concordant treatment, and strengthen regional and state-level capacity in high-burden groups so that progress in colorectal cancer control is shared more equitably nationwide. Practical strategies include expanding organized FIT-based screening and colonoscopy capacity, implementing culturally tailored outreach and patient navigation for underrepresented racial and ethnic groups and younger adults, and strengthening data integration across registries, claims, and clinical information systems to identify and close gaps along the diagnostic and treatment continuum. Because AAMIR is a population-level metric derived from separate incidence and mortality registries rather than a direct measure of individual survival, it should not be interpreted as estimating survival probabilities. Instead, these findings should be interpreted as contextual signals of how effectively reductions in disease occurrence translate into mortality gains across groups and as descriptive guidance for prioritizing future cancer-control efforts.

## Figures and Tables

**Table 1:** Age-adjusted incidence rates (AAIR), mortality rates (AAMR), and mortality-to-incidence ratios (AAMIR) for colorectal cancer in the United States, 1999–2021, by sex, age group, race/ethnicity, and geographic region.

Subgroup	AAIR (1999)	AAIR (2021)	AAPC AAIR (95% CI)	AAMR (1999)	AAMR (2021)	AAPC AAMR (95% CI)	AAMIR (1999)	AAMIR (2021)	AAPC AAMIR (95% CI)
**Overall**	78.24	50.79	−2.20 (−2.52, −1.89) *	29.34	17.92	−2.33 (−2.46, −2.20) *	0.375	0.353	−0.08 (−0.47, 0.30)
**Sex**									
Male	93.48	57.32	−2.44 (−2.76, −2.13) *	35.72	21.36	−2.36 (−2.65, −2.08) *	0.382	0.373	0.07 (−0.09, 0.24)
Female	67.04	45.02	−2.06 (−2.38, −1.74) *	24.89	15.04	−2.40 (−2.55, −2.26) *	0.371	0.334	−0.29 (−0.43, −0.15) *
**Age Group**									
20–39 years	3.68	6.45	2.42 (1.76, 3.08) *	0.92	1.14	0.87 (0.63, 1.11) *	0.249	0.177	−1.71 (−2.85, −0.56) *
40–65 years	52.63	51.15	−0.39 (−0.72, −0.05) *	15.51	13.06	−0.82 (−1.28, −0.36) *	0.295	0.255	−0.48 (−1.20, 0.25)
≥65 years	308.13	154.22	−3.44 (−3.61, −3.27) *	126.23	67.17	−2.98 (−3.12, −2.85) *	0.410	0.436	0.48 (−0.28, 1.24)
**Race/Ethnicity**									
Non-Hispanic White	78.68	51.66	−2.11 (−2.40, −1.82) *	29.01	17.97	−2.22 (−2.50, −1.93) *	0.368	0.347	−0.11% (−0.25 to +0.03)
Non-Hispanic Black	87.53	57.88	−2.05 (−2.50, −1.61) *	40.4	23.16	−2.67 (−2.77, −2.57) *	0.462	0.400	−0.54 (−0.90, −0.18) *
Hispanic	64.54	47.47	−1.73 (−1.96, −1.50) *	20.28	14.97	−1.51 (−1.62, −1.40) *	0.314	0.315	0.28 (−0.48, 1.05)
Non-Hispanic Asian/Pacific Islander	59.44	41.55	−2.07 (−2.31, −1.82) *	16.99	13.08	−1.73 (−1.95, −1.50) *	0.286	0.315	0.81 (−0.64, 2.28)
Non-Hispanic American Indian/Alaska Native	67.54	62.11	−0.27 (−0.58, 0.04)	21.36	18.19	−0.84 (−1.36, −0.32) *	0.316	0.293	−0.43 (−0.85, 0.01)
**Region**									
Northeast	86.56	50.62	−2.73 (−3.05, −2.42) *	32.10	15.86	−3.21 (−3.38, −3.04) *	0.371	0.313	−0.40 (−0.60, −0.20) *
Midwest	82.21	53.34	−2.16 (−2.52, −1.79) *	31.12	18.86	−2.36 (−2.51, −2.20) *	0.379	0.354	−0.15 (−0.33, 0.04)
South	75.64	53.72	−1.83 (−2.18, −1.48) *	28.62	18.96	−2.01 (−2.15, −1.87) *	0.378	0.353	−0.14 (−0.29, 0.01)
West	70.67	47.63	−2.00 (−2.31, −1.70) *	25.52	16.96	−2.01 (−2.19, −1.84) *	0.361	0.356	0.02 (−0.13, 0.17)

Notes: AAPC—average annual percent change; CI—confidence interval; *—statistically significant (*p* < 0.05). Rates per 100,000 (2000 U.S. standard population). AAMIR data for Non-Hispanic White was not provided in the results. Full segmented APCs are available in Supplemental Tables. Census Bureau regions are defined as follows: Northeast: Connecticut, Maine, Massachusetts, New Hampshire, Rhode Island, Vermont, New Jersey, New York, and Pennsylvania. Midwest: Illinois, Indiana, Michigan, Ohio, Wisconsin, Iowa, Kansas, Minnesota, Missouri, Nebraska, North Dakota, and South Dakota. South: Delaware, District of Columbia, Florida, Georgia, Maryland, North Carolina, South Carolina, Virginia, West Virginia, Alabama, Kentucky, Mississippi, Tennessee, Arkansas, Louisiana, Oklahoma, and Texas. West: Arizona, Colorado, Idaho, Montana, Nevada, New Mexico, Utah, Wyoming, Alaska, California, Hawaii, Oregon, and Washington.

## Data Availability

The original data presented in this study are openly available in the Centers for Disease Control and Prevention (CDC) WONDER United States Cancer Statistics (USCS) database at https://wonder.cdc.gov/cancer.html, accessed on 30 September 2025. All analyses were performed on publicly accessible, de-identified aggregate data; no restricted or individual-level data were used.

## Supplementary Materials

The following supporting information can be downloaded at: https://www.mdpi.com/article/10.3390/diseases13120392/s1, Supplemental Table S1: Joinpoint results for age-adjusted mortality-to-incidence ratio (AAMIR), 1999–2021; Supplemental Table S2: Joinpoint results for age-adjusted incidence rate (AAIR), United States, 1999–2021; Supplemental Table S3: Joinpoint results for age-adjusted mortality rate (AAMR), United States, 1999–2021; Supplemental Table S4: Annual Age-Adjusted Incidence Rates (AAIR), Mortality Rates (AAMR), Incidence Counts, Death Counts, Populations, and Mortality-to-Incidence Ratios (AAMIR) for Colorectal Cancer, United States, 1999–2021 (Overall); Supplemental Table S5: Annual Age-Adjusted Incidence Rates (AAIR), Mortality Rates (AAMR), Incidence Counts, Death Counts, Populations, and Mortality-to-Incidence Ratios (AAMIR) for Colorectal Cancer, United States, 1999–2021 (Male Trends and Female Trends); Supplemental Table S6: Joinpoint Regression Results for Colorectal Cancer Trends by Age Group, 1999–2021; Supplemental Table S7: Annual Age-Adjusted Incidence Rates (AAIR), Mortality Rates (AAMR), and Mortality-to-Incidence Ratios (AAMIR) for Colorectal Cancer by Race/Ethnicity, 1999–2021; Supplemental Table S8: Annual Age-Adjusted Incidence Rates (AAIR), Mortality Rates (AAMR), and Mortality-to-Incidence Ratios (AAMIR) for Colorectal Cancer by Geographic Region, 1999–2021; Supplemental Table S9: State-Level AAMIR Trends for Colorectal Cancer, 1999–2021 (Top 5 Highest and Lowest AAMIR States in 2021); Supplemental Figure S1: a U.S. state-level choropleth map of 2021 AAMIR values.
