# Supplementary material for: Colorectal Cancer in the U.S., 1999–2021: Declining Rates, Rising Concerns, and Persistent Disparities
Source: Diseases. 2025 Dec 4;13(12):392. doi: 10.3390/diseases13120392 (PMC12731979; doi:10.3390/diseases13120392)

Supplemental Table 1. Joinpoint results for age-adjusted mortality-to-incidence ratio (AAMIR), 1999–2021

Subgroup	Joinpoints (n)	Joinpoint years	AAPC % (95% CI)	p-value
Overall	3	2005, 2012, 2018	−0.08 (−0.47, 0.30)	0.669
Males	0	–	0.07 (−0.09, 0.24)	0.353
Females	0	–	−0.29 (−0.43, −0.15)*	<0.001
Age 20–39 yr	3	2005, 2012, 2017	−1.71 (−2.85, −0.56)*	0.004
Age 40–65 yr	3	2005, 2011, 2015	−0.48 (−1.20, 0.25)	0.196
Age ≥65 yr	3	2005, 2015, 2018	0.48 (−0.28, 1.24)	0.215
Non-Hispanic Black	1	2004	−0.54 (−0.90, −0.18)*	0.003
Northeast	0	–	−0.40 (−0.60, −0.20)*	<0.001
States with unfavorable AAMIR trend				
Nebraska	3	2005, 2013, 2016	0.51 (−1.22, 2.27)	0.564
Arizona	0	–	0.28 (0.02, 0.54)*	0.035
Oklahoma	0	–	0.37 (0.02, 0.73)*	0.040
States with favorable AAMIR trend				
New Jersey	0	–	−0.64 (−0.93, −0.35)*	<0.001
Massachusetts	0	–	−0.50 (−0.79, −0.22)*	0.002

- $p < 0.05$

Supplemental Table 2. Joinpoint results for age-adjusted incidence rate (AAIR), United States, 1999–2021

Subgroup	Joinpoints (n)	Joinpoint years	AAPC % (95% CI)	p-value
Overall	1	2012	–2.20 (–2.52, –1.89)*	<0.001
Males	1	2012	–2.44 (–2.76, –2.13)*	<0.001
Females	1	2012	–2.06 (–2.38, –1.74)*	<0.001
Age 20–39 years	2	2013, 2016	2.42 (1.76, 3.08)*	<0.001
Age 40–65 years	1	2011	–0.39 (–0.72, –0.05)*	0.024
Age ≥65 years	0	–	–3.44 (–3.61, –3.27)*	<0.001
Non-Hispanic White	1	2012	–2.11 (–2.40, –1.82)*	<0.001
Non-Hispanic Black	1	2003	–2.05 (–2.50, –1.61)*	<0.001
Hispanic	0	–	–1.73 (–1.96, –1.50)*	<0.001
Non-Hispanic Asian/PI	0	–	–2.07 (–2.31, –1.82)*	<0.001
Non-Hispanic AI/AN	0	–	–0.27 (–0.58, 0.04)	0.083
Northeast	1	2012	–2.73 (–3.05, –2.42)*	<0.001
Midwest	1	2012	–2.16 (–2.52, –1.79)*	<0.001
South	1	2012	–1.83 (–2.18, –1.48)*	<0.001
West	1	2013	–2.00 (–2.31, –1.70)*	<0.001
Selected high-burden states (example)				
Nebraska	3	2007, 2013, 2016	–2.33 (–3.86, –0.78)*	0.003
Arizona	0	–	–2.02 (–2.24, –1.80)*	<0.001
Oklahoma	0	–	–1.53 (–1.81, –1.25)*	<0.001
Selected low-burden states (example)				
New Jersey	1	2012	–2.55 (–2.95, –2.14)*	<0.001

Subgroup	Joinpoints (n)	Joinpoint years	AAPC % (95% CI)	p-value
Massachusetts	2	2002, 2010	–2.85 (–3.51, –2.18)*	<0.001

- p < 0.05 AI/AN = American Indian/Alaska Native; PI = Pacific Islander

Supplemental Table 3. Joinpoint results for age-adjusted mortality rate (AAMR), United States, 1999–2021

Subgroup	Joinpoints (n)	Joinpoint years	AAPC % (95% CI)	p-value
Overall	1	2011	–2.33 (–2.46, –2.20)*	<0.001
Males	3	2002, 2005, 2012	–2.36 (–2.65, –2.08)*	<0.001
Females	1	2010	–2.40 (–2.55, –2.26)*	<0.001
Age 20–39 years	0	–	0.87 (0.63, 1.11)*	<0.001
Age 40–65 years	2	2002, 2005	–0.82 (–1.28, –0.36)*	0.001
Age ≥65 years	1	2012	–2.98 (–3.12, –2.85)*	<0.001
Non-Hispanic White	3	2002, 2005, 2012	–2.22 (–2.50, –1.93)*	<0.001
Non-Hispanic Black	0	–	–2.67 (–2.77, –2.57)*	<0.001
Hispanic	0	–	–1.51 (–1.62, –1.40)*	<0.001
Non-Hispanic Asian/PI	0	–	–1.73 (–1.95, –1.50)*	<0.001
Non-Hispanic AI/AN	0	–	–0.84 (–1.36, –0.32)*	0.003
Northeast	1	2009	–3.21 (–3.38, –3.04)*	<0.001
Midwest	1	2010	–2.36 (–2.51, –2.20)*	<0.001
South	1	2011	–2.01 (–2.15, –1.87)*	<0.001
West	1	2012	–2.01 (–2.19, –1.84)*	<0.001
Selected high-burden states (example)				
Nebraska	1	2017	–1.75 (–2.44, –1.06)*	0.000
Arizona	1	2009	–1.80 (–2.24, –1.35)*	<0.001
Oklahoma	0	–	–1.18 (–1.46, –0.90)*	<0.001
Selected low-burden states (example)				
New Jersey	0	–	–3.28 (–3.44, –3.11)*	<0.001

Subgroup	Joinpoints (n)	Joinpoint years	AAPC % (95% CI)	p-value
Massachusetts	1	2012	–3.70 (–4.08, –3.33)*	<0.001

- $p < 0.05$ AI/AN = American Indian/Alaska Native; PI = Pacific Islander

Supplemental Table 4: Annual Age-Adjusted Incidence Rates (AAIR), Mortality Rates (AAMR), Incidence Counts, Death Counts, Populations, and Mortality-to-Incidence Ratios (AAMIR) for Colorectal Cancer, United States, 1999–2021 (Overall)

Year	Incidence Count	Population	AAIR	AAIR Lower CI	AAIR Upper CI	AAIR SE	Death Count	Death Population	AAMR	AAMR Lower CI	AAMR Upper CI	AAMR SE	AAMIR
1999	150908	196524362	78.238	77.844	78.634	0.201	57208	199000198	29.343	29.103	29.585	0.123	0.375
2000	152574	199083271	77.859	77.469	78.251	0.199	57417	201587615	28.973	28.736	29.211	0.121	0.372
2001	153034	202073230	76.746	76.362	77.132	0.196	56796	204062414	28.26	28.028	28.493	0.119	0.368
2002	151804	204449149	74.862	74.486	75.24	0.192	56592	206451793	27.703	27.475	27.933	0.117	0.370
2003	153208	208682117	73.505	73.137	73.874	0.188	55769	208682117	26.842	26.619	27.066	0.114	0.365
2004	151368	211050944	71.393	71.033	71.755	0.184	53565	211050944	25.367	25.152	25.583	0.11	0.355
2005	150132	213371926	69.497	69.145	69.851	0.18	52987	213371926	24.633	24.423	24.844	0.107	0.354
2006	148303	216055494	67.392	67.048	67.738	0.176	53187	216055494	24.23	24.023	24.437	0.106	0.360
2007	147713	218481776	65.726	65.389	66.065	0.172	53205	218481776	23.703	23.501	23.906	0.103	0.361
2008	146987	220975702	64.075	63.745	64.406	0.169	50988	220975702	23.074	22.877	23.273	0.101	0.360
2009	143102	223491138	61.118	60.798	61.439	0.163	49526	223491138	22.16	21.968	22.353	0.098	0.363
2010	139565	226545566	58.368	58.059	58.679	0.158	49287	226545566	21.756	21.567	21.946	0.096	0.373
2011	138090	229472164	57.296	56.991	57.602	0.156	48710	229472164	21.227	21.042	21.413	0.094	0.370
2012	135062	232164325	55.716	55.418	56.015	0.152	47988	232164325	20.67	20.49	20.852	0.092	0.371
2013	133387	234721973	55.094	54.8	55.389	0.15	47855	234721973	20.388	20.21	20.567	0.091	0.370
2014	133289	237174896	55.004	54.713	55.296	0.149	47222	237174896	19.91	19.736	20.085	0.089	0.362
2015	132132	239618116	54.496	54.209	54.785	0.147	47392	239618116	19.778	19.606	19.951	0.088	0.363
2016	130579	242091773	53.844	53.559	54.129	0.145	46808	242091773	19.335	19.166	19.504	0.086	0.359
2017	128406	244539437	52.811	52.532	53.091	0.143	46639	244539437	19.072	18.906	19.239	0.085	0.361
2018	127406	246819201	52.439	52.163	52.716	0.141	45908	246819201	18.6	18.437	18.763	0.083	0.355
2019	127499	249300671	52.256	51.982	52.531	0.14	45253	249300671	18.152	17.993	18.312	0.081	0.347

Year	Incidence Count	Population	AAIR	AAIR Lower CI	AAIR Upper CI	AAIR SE	Death Count	Death Population	AAMR	AAMR Lower CI	AAMR Upper CI	AAMR SE	AAMIR
2020	114008	251525264	46.52	46.262	46.778	0.132	44862	251525264	17.836	17.68	17.994	0.08	0.383
2021	121371	254036372	50.788	50.52	51.057	0.137	45531	254036372	17.923	17.768	18.08	0.08	0.353

Supplemental Table 5: Annual Age-Adjusted Incidence Rates (AAIR), Mortality Rates (AAMR), Incidence Counts, Death Counts, Populations, and Mortality-to-Incidence Ratios (AAMIR) for Colorectal Cancer, United States, 1999–2021 (Male Trends and Female Trends)

Year	Incidence Count	Population	AAIR	AAIR Lower CI	AAIR Upper CI	AAIR SE	Death Count	Death Population	AAMR	AAMR Lower CI	AAMR Upper CI	AAMR SE	AAMIR
1999	74704	101918297	67.037	66.555	67.522	0.247	28902	103221707	24.888	24.6	25.178	0.147	0.371
2000	75180	103133651	66.556	66.079	67.036	0.244	28944	104448849	24.529	24.245	24.815	0.145	0.369
2001	75568	104610919	65.968	65.497	66.443	0.241	28574	105661358	24	23.72	24.281	0.143	0.364
2002	75171	106843948	64.367	63.904	64.833	0.237	28129	106843948	23.313	23.039	23.588	0.14	0.362
2003	74902	108164000	63.228	62.774	63.685	0.232	27591	108164000	22.701	22.433	22.971	0.137	0.359
2004	72845	109498000	61.542	61.096	61.99	0.228	26199	109498000	21.501	21.242	21.763	0.133	0.349
2005	72134	110835000	60.168	59.731	60.609	0.224	25470	110835000	20.797	20.544	21.053	0.13	0.346
2006	70946	112175000	58.908	58.478	59.341	0.22	25387	112175000	20.649	20.398	20.902	0.128	0.351
2007	69456	113518000	57.086	56.665	57.51	0.215	24953	113518000	20.131	19.885	20.379	0.126	0.353
2008	67926	114864000	55.67	55.257	56.085	0.211	24485	114864000	19.591	19.35	19.835	0.123	0.352
2009	66192	116213000	53.336	52.934	53.74	0.207	23389	116213000	18.618	18.385	18.854	0.119	0.349
2010	64645	117565000	51.106	50.715	51.5	0.202	22968	117565000	18.219	17.99	18.451	0.117	0.356
2011	63140	118920000	49.867	49.48	50.256	0.199	22367	118920000	17.947	17.722	18.174	0.115	0.360
2012	61788	120278000	48.704	48.323	49.088	0.196	21742	120278000	17.415	17.194	17.638	0.112	0.358
2013	60746	121639000	48.139	47.764	48.517	0.193	21349	121639000	17.105	16.888	17.325	0.11	0.355
2014	60115	123003000	48.196	47.826	48.569	0.189	20958	123003000	16.782	16.569	16.997	0.108	0.348

**Year**	**Incidence Count**	**Population**	**AAIR**	**AAIR Lower CI**	**AAIR Upper CI**	**AAIR SE**	**Death Count**	**Death Population**	**AAMR**	**AAMR Lower CI**	**AAMR Upper CI**	**AAMR SE**	**AAMIR**
2015	59809	124370000	47.711	47.345	48.08	0.187	20826	124370000	16.745	16.533	16.959	0.107	0.351
2016	59579	125740000	47.82	47.456	48.187	0.186	20516	125740000	16.316	16.108	16.526	0.105	0.341
2017	58620	127113000	46.469	46.112	46.83	0.183	20511	127113000	16.136	15.93	16.344	0.104	0.347
2018	57989	128489000	46.058	45.704	46.414	0.181	19938	128489000	15.517	15.316	15.72	0.102	0.337
2019	57866	129868000	45.866	45.515	46.22	0.179	19836	129868000	15.274	15.076	15.474	0.1	0.333
2020	52000	131250000	41.104	40.772	41.439	0.169	19488	131250000	14.848	14.652	15.046	0.1	0.361
2021	58685	132634000	45.016	44.653	45.383	0.186	19951	132634000	15.042	14.835	15.252	0.105	0.334

Male Trends

**Year**	**Incidence Count**	**Population**	**AAIR**	**AAIR Lower CI**	**AAIR Upper CI**	**AAIR SE**	**Death Count**	**Death Population**	**AAMR**	**AAMR Lower CI**	**AAMR Upper CI**	**AAMR SE**	**AAMIR**
1999	76104	94506065	93.479	92.846	94.115	0.324	28306	95778491	35.718	35.346	36.092	0.191	0.382
2000	77394	95849620	92.901	92.274	93.531	0.32	28473	97138766	35.158	34.792	35.527	0.187	0.378
2001	77466	97362195	91.009	90.392	91.63	0.313	28222	98401056	34.168	33.809	34.53	0.183	0.375
2002	76633	97505045	88.666	88.06	89.275	0.307	28463	99607845	33.654	33.301	34.011	0.18	0.380
2003	78206	100518000	86.936	86.337	87.539	0.303	28178	100518000	32.492	32.148	32.839	0.176	0.374
2004	78523	101553000	84.099	83.511	84.69	0.299	27366	101553000	30.575	30.239	30.914	0.172	0.364
2005	77998	102536000	81.373	80.797	81.952	0.293	26517	102536000	29.763	29.435	30.094	0.168	0.366
2006	77357	103480000	78.076	77.514	78.641	0.285	27800	103480000	29.014	28.692	29.338	0.165	0.372
2007	78257	104964000	76.467	75.915	77.023	0.282	28252	104964000	28.365	28.048	28.684	0.162	0.371
2008	79061	109111000	74.451	73.912	74.993	0.276	26503	109111000	27.529	27.218	27.843	0.159	0.370
2009	76910	107278000	70.673	70.153	71.194	0.265	26137	107278000	26.728	26.422	27.036	0.156	0.378
2010	74920	108981000	67.301	66.8	67.805	0.256	26319	108981000	26.297	26	26.597	0.153	0.391

Year	Incidence Count	Population	AAIR	AAIR Lower CI	AAIR Upper CI	AAIR SE	Death Count	Death Population	AAMR	AAMR Lower CI	AAMR Upper CI	AAMR SE	AAMIR
2011	74950	110552000	66.307	65.814	66.803	0.252	26343	110552000	25.384	25.075	25.697	0.158	0.383
2012	73274	111886000	64.125	63.646	64.605	0.244	26246	111886000	24.757	24.455	25.061	0.154	0.386
2013	72641	113082000	63.39	62.92	63.862	0.24	26506	113082000	24.478	24.181	24.777	0.152	0.386
2014	73174	114172000	63.13	62.666	63.596	0.237	26264	114172000	23.813	23.524	24.105	0.148	0.377
2015	72323	116248000	62.46	62.003	62.92	0.234	26566	116248000	23.539	23.255	23.826	0.146	0.377
2016	70999	116352000	60.893	60.446	61.342	0.228	26292	116352000	23.047	22.769	23.328	0.142	0.378
2017	69786	117426000	60.106	59.666	60.547	0.225	26128	117426000	22.658	22.385	22.933	0.139	0.377
2018	69417	118330000	59.78	59.346	60.216	0.222	25970	118330000	22.362	22.094	22.633	0.137	0.374
2019	69633	119433000	59.557	59.128	59.989	0.219	25417	119433000	21.597	21.337	21.86	0.133	0.363
2020	62008	120275000	52.677	52.276	53.081	0.205	25374	120275000	21.45	21.193	21.709	0.131	0.407
2021	62686	121402000	57.316	56.9	57.736	0.213	25927	121402000	21.356	21.101	21.613	0.13	0.373

Supplemental Table 6: Joinpoint Regression Results for Colorectal Cancer Trends by Age Group, 1999–2021

Age Group	Metric	Period	APC (95% CI)	p-value
20–39 years	AAIR	1999–2013	2.17 (1.91, 2.43)	<0.001*
20–39 years	AAIR	2013–2016	6.45 (1.55, 11.60)	0.013*
20–39 years	AAIR	2016–2021	0.76 (-0.21, 1.74)	0.118
20–39 years	AAMR	1999–2021	0.87 (0.63, 1.11)	<0.001*
20–39 years	AAMIR	1999–2005	-2.82 (-4.57, -1.04)	0.005*
20–39 years	AAMIR	2005–2012	0.02 (-1.73, 1.81)	0.976
20–39 years	AAMIR	2012–2017	-4.70 (-8.05, -1.23)	0.012*
20–39 years	AAMIR	2017–2021	0.79 (-2.89, 4.60)	0.654
40–65 years	AAIR	1999–2011	-1.17 (-1.62, -0.72)	<0.001*
40–65 years	AAIR	2011–2021	0.56 (-0.02, 1.14)	0.056
40–65 years	AAMR	1999–2002	-1.10 (-2.71, 0.54)	0.172
40–65 years	AAMR	2002–2005	-4.12 (-7.21, -0.93)	0.015*
40–65 years	AAMR	2005–2021	-0.14 (-0.26, -0.02)	0.028*
40–65 years	AAMIR	1999–2005	-2.21 (-3.17, -1.24)	<0.001*
40–65 years	AAMIR	2005–2011	1.41 (0.10, 2.74)	0.037*
40–65 years	AAMIR	2011–2015	-1.78 (-5.05, 1.61)	0.272
40–65 years	AAMIR	2015–2021	0.27 (-0.72, 1.27)	0.565

Age Group	Metric	Period	APC (95% CI)	p-value
≥65 years	AAIR	1999–2021	-3.44 (-3.61, -3.27)	<0.001*
≥65 years	AAMR	1999–2012	-3.28 (-3.43, -3.13)	<0.001*
≥65 years	AAMR	2012–2021	-2.55 (-2.84, -2.27)	<0.001*
≥65 years	AAMIR	1999–2005	-0.30 (-0.89, 0.29)	0.287
≥65 years	AAMIR	2005–2015	0.82 (0.53, 1.11)	<0.001*
≥65 years	AAMIR	2015–2018	-0.90 (-6.22, 4.73)	0.729
≥65 years	AAMIR	2018–2021	2.30 (0.06, 4.59)	0.045*

Supplemental Table 7: Annual Age-Adjusted Incidence Rates (AAIR), Mortality Rates (AAMR), and Mortality-to-Incidence Ratios (AAMIR) for Colorectal Cancer by Race/Ethnicity, 1999–2021

Non-Hispanic American Indian/Alaska Native Trends

Year	AAIR	AAMR	AAMIR
1999	67.544	21.363	0.316
2000	59.349	21.583	0.364
2001	63.22	20.601	0.326
2002	60.651	24.449	0.403
2003	62.907	20.507	0.326
2004	59.887	21.346	0.356
2005	59.523	21.181	0.356
2006	63.21	19.358	0.306
2007	63.03	21.06	0.334
2008	61.84	25.386	0.411
2009	60.351	23.402	0.388
2010	60.486	20.622	0.341
2011	60.236	22.553	0.374
2012	58.625	20.434	0.349
2013	60.148	23.28	0.387
2014	60.925	20.39	0.335
2015	63.077	21.349	0.338
2016	64.355	19.044	0.296
2017	62.249	20.657	0.332
2018	59.216	19.029	0.321
2019	58.024	17.76	0.306
2020	52.614	18.734	0.356
2021	62.112	18.193	0.293

Non-Hispanic Asian or Pacific Islander Trends

Year	AAIR	AAMR	AAMIR
1999	59.44	16.987	0.286
2000	59.516	17.726	0.298
2001	59.266	18.703	0.316
2002	59.598	17.949	0.301

Year	AAIR	AAMR	AAMIR
2003	55.66	17.473	0.314
2004	54.98	16.263	0.296
2005	54.084	16.311	0.302
2006	53.564	15.923	0.297
2007	51.871	15.808	0.305
2008	51.683	16.38	0.317
2009	49.585	14.865	0.3
2010	49.098	15.839	0.323
2011	47.957	15.222	0.317
2012	45.102	15.391	0.341
2013	44.205	13.996	0.317
2014	44.668	13.798	0.309
2015	42.646	14.143	0.332
2016	43.541	13.282	0.305
2017	42.176	13.321	0.316
2018	41.147	12.64	0.307
2019	41.562	12.811	0.308
2020	35.346	12.568	0.356
2021	41.548	13.081	0.315

Non-Hispanic Black Trends

Year	AAIR	AAMR	AAMIR
1999	87.53	40.404	0.462
2000	87.304	40.094	0.459
2001	86.508	39.504	0.457
2002	87.02	38.596	0.444
2003	88.197	37.976	0.431
2004	85.184	35.674	0.419
2005	83.282	35.869	0.431
2006	81.731	34.91	0.427
2007	79.633	33.706	0.423
2008	78.812	32.672	0.415
2009	75.856	31.435	0.414
2010	72.388	30.925	0.427
2011	69.674	30.367	0.436
2012	67.391	28.619	0.425
2013	66.713	27.917	0.418
2014	65.61	26.744	0.408
2015	64.467	27.034	0.419
2016	62.9	26.614	0.423
2017	60.429	25.759	0.426
2018	60.846	24.457	0.402
2019	59.817	23.822	0.398
2020	52.769	23.665	0.448

Year	AAIR	AAMR	AAMIR
2021	57.875	23.16	0.4

Non-Hispanic White Trends

Year	AAIR	AAMR	AAMIR
1999	78.678	29.006	0.369
2000	78.448	28.64	0.365
2001	77.232	27.867	0.361
2002	75.057	27.337	0.364
2003	73.432	26.397	0.359
2004	71.236	25.029	0.351
2005	69.32	24.168	0.349
2006	67.006	23.79	0.355
2007	65.463	23.35	0.357
2008	63.692	22.66	0.356
2009	60.613	21.779	0.359
2010	57.92	21.353	0.369
2011	56.937	20.848	0.366
2012	55.726	20.373	0.366
2013	55.031	20.145	0.366
2014	55.151	19.781	0.359
2015	54.917	19.577	0.356
2016	54.276	19.194	0.354
2017	53.527	18.942	0.354
2018	53.083	18.581	0.35
2019	53.245	18.18	0.341
2020	47.744	17.805	0.373
2021	51.659	17.967	0.348

Hispanic Trends

Year	AAIR	AAMR	AAMIR
1999	64.535	20.276	0.314
2000	64.216	19.681	0.306
2001	63.749	20.116	0.316
2002	62.261	19.832	0.319
2003	61.549	19.447	0.316
2004	62.195	18.58	0.299
2005	60.168	18.512	0.308
2006	59.64	18.879	0.317
2007	57.572	17.971	0.312
2008	56.558	17.777	0.314
2009	54.599	17.778	0.326
2010	51.57	16.967	0.329
2011	51.851	16.806	0.324

Year	AAIR	AAMR	AAMIR
2012	49.438	16.597	0.336
2013	49.712	16.634	0.335
2014	49.671	15.911	0.32
2015	49.43	15.664	0.317
2016	48.843	15.351	0.314
2017	48.345	15.646	0.324
2018	48.234	15.486	0.321
2019	47.204	14.815	0.314
2020	41.766	14.799	0.354
2021	47.468	14.971	0.315

Supplemental Table 8: Annual Age-Adjusted Incidence Rates (AAIR), Mortality Rates (AAMR), and Mortality-to-Incidence Ratios (AAMIR) for Colorectal Cancer by Geographic Region, 1999–2021

Northeast Trends

Year	AAIR	AAMR	AAMIR
1999	86.559	32.098	0.371
2000	85.025	31.325	0.368
2001	84.673	30.111	0.356
2002	82.228	29.453	0.358
2003	78.472	27.991	0.357
2004	76.317	26.28	0.344
2005	73.69	25.064	0.340
2006	70.451	24.656	0.350
2007	68.471	23.839	0.348
2008	65.775	23.132	0.352
2009	63.278	22.166	0.350
2010	60.163	21.766	0.362
2011	58.948	20.542	0.348
2012	56.789	20.28	0.357
2013	56.033	19.704	0.352
2014	55.184	18.886	0.342
2015	54.365	18.732	0.345
2016	53.672	18.286	0.341
2017	52.349	17.899	0.342
2018	50.897	17.099	0.336
2019	51.76	16.367	0.316
2020	45.859	16.372	0.357
2021	50.623	15.855	0.313

Midwest Trends

Year	AAIR	AAMR	AAMIR
1999	82.207	31.117	0.379
2000	81.193	30.043	0.370
2001	80.288	29.411	0.366
2002	78.762	28.86	0.366
2003	77.077	28.008	0.363
2004	74.522	26.399	0.354
2005	72.755	25.525	0.351
2006	71.865	25.381	0.353
2007	69.597	24.619	0.354
2008	67.802	24.099	0.355
2009	63.944	22.885	0.358
2010	61.311	22.687	0.370
2011	60.44	22.237	0.368
2012	58.585	21.373	0.365
2013	57.923	21.299	0.368
2014	58.094	20.82	0.358
2015	58.036	20.543	0.354
2016	58.032	19.972	0.344
2017	56.458	19.802	0.351
2018	56.075	19.57	0.349
2019	54.764	18.779	0.343
2020	48.452	18.616	0.384
2021	53.335	18.861	0.354

South Trends

Year	AAIR	AAMR	AAMIR
1999	75.635	28.624	0.378
2000	76.791	28.928	0.377
2001	75.359	28.219	0.374
2002	73.604	27.802	0.378
2003	73.322	26.815	0.366
2004	71.862	25.663	0.357
2005	69.677	25.035	0.359
2006	67.351	24.64	0.366
2007	66	24.276	0.368
2008	64.069	23.731	0.370
2009	61.163	22.77	0.372
2010	58.599	22.245	0.380
2011	57.951	21.806	0.376
2012	56.995	21.344	0.374
2013	56.591	21.152	0.374
2014	56.738	20.796	0.367
2015	56.677	20.697	0.365
2016	55.856	20.376	0.365

Year	AAIR	AAMR	AAMIR
2017	54.821	20.046	0.366
2018	55.388	19.645	0.355
2019	55.158	19.333	0.351
2020	49.264	18.808	0.382
2021	53.723	18.957	0.353

West Trends

Year	AAIR	AAMR	AAMIR
1999	70.665	25.521	0.361
2000	69.74	25.263	0.362
2001	68.163	24.998	0.367
2002	66.36	24.343	0.367
2003	66.164	24.333	0.368
2004	63.615	22.683	0.357
2005	62.883	22.37	0.356
2006	60.961	21.703	0.356
2007	59.993	21.454	0.358
2008	59.874	20.716	0.346
2009	57.538	20.222	0.351
2010	54.628	19.808	0.363
2011	53.04	19.721	0.372
2012	51.285	19.034	0.371
2013	50.41	18.652	0.370
2014	50.664	18.304	0.361
2015	49.602	18.298	0.369
2016	48.933	17.801	0.364
2017	48.662	17.69	0.364
2018	48.464	17.116	0.353
2019	48.393	16.991	0.351
2020	43.691	16.619	0.380
2021	47.627	16.963	0.356

Supplemental Table 9: State-Level AAMIR Trends for Colorectal Cancer, 1999–2021 (Top 5 Highest and Lowest AAMIR States in 2021)

State	AAMIR (1999)	AAMIR (2021)	AAPC AAMIR (95% CI)	AAIR (1999)	AAIR (2021)	AAPC AAIR (95% CI)	AAMR (1999)	AAMR (2021)	AAPC AAMR (95% CI)
**Highest AAMIR States**									
Nebraska	0.364	0.402	0.51 (-1.22, 2.27)	85.78	52.95	-2.33 (-3.86, -0.78)*	31.23	21.26	-1.75 (-2.44, -1.06)*

State	AAMIR (1999)	AAMIR (2021)	AAPC AAMIR (95% CI)	AAIR (1999)	AAIR (2021)	AAPC AAIR (95% CI)	AAMR (1999)	AAMR (2021)	AAPC AAMR (95% CI)
Vermont	0.367	0.352	0.40 (-0.36, 1.16)	83.93	46.75	-2.87 (-3.25, -2.50)*	30.79	16.44	-2.47 (-3.04, -1.89)*
Oklahoma	0.387	0.371	0.37 (0.02, 0.73)*	75.63	60.75	-1.53 (-1.81, -1.25)*	29.28	22.55	-1.18 (-1.46, -0.90)*
Arizona	0.375	0.402	0.28 (0.02, 0.54)*	65.54	42.95	-2.02 (-2.24, -1.80)*	24.57	17.27	-1.80 (-2.24, -1.35)*
Rhode Island	0.347	0.324	0.27 (-0.24, 0.78)	94.31	44.47	-3.36 (-4.09, -2.62)*	32.75	14.43	-3.46 (-3.84, -3.08)*
**Lowest AAMIR States**									
New Jersey	0.369	0.292	-0.64 (-0.93, -0.35)*	89.12	55.72	-2.55 (-2.95, -2.14)*	32.87	16.27	-3.28 (-3.44, -3.11)*
New Hampshire	0.423	0.319	-0.63 (-1.19, -0.05)*	76.70	46.63	-2.69 (-3.13, -2.24)*	32.46	14.87	-3.32 (-3.79, -2.85)*
Massachusetts	0.386	0.304	-0.50 (-0.79, -0.22)*	81.80	45.51	-2.85 (-3.51, -2.18)*	31.58	13.86	-3.70 (-4.08, -3.33)*
Maryland	0.403	0.369	-0.44 (-0.70, -0.17)*	78.01	49.23	-2.31 (-2.80, -1.81)*	31.46	18.14	-2.80 (-3.17, -2.42)*
Minnesota	0.359	0.306	-0.43 (-0.75, -0.11)*	73.37	52.52	-1.98 (-2.23, -1.73)*	26.32	16.05	-2.44 (-2.73, -2.14)*

Notes: Rates per 100,000 (2000 U.S. standard population). * = statistically significant ($p < 0.05$). For full annual state-level data, refer to the source files.

Supplemental Figure 1: a U.S. state-level choropleth map of 2021 AAMIR values

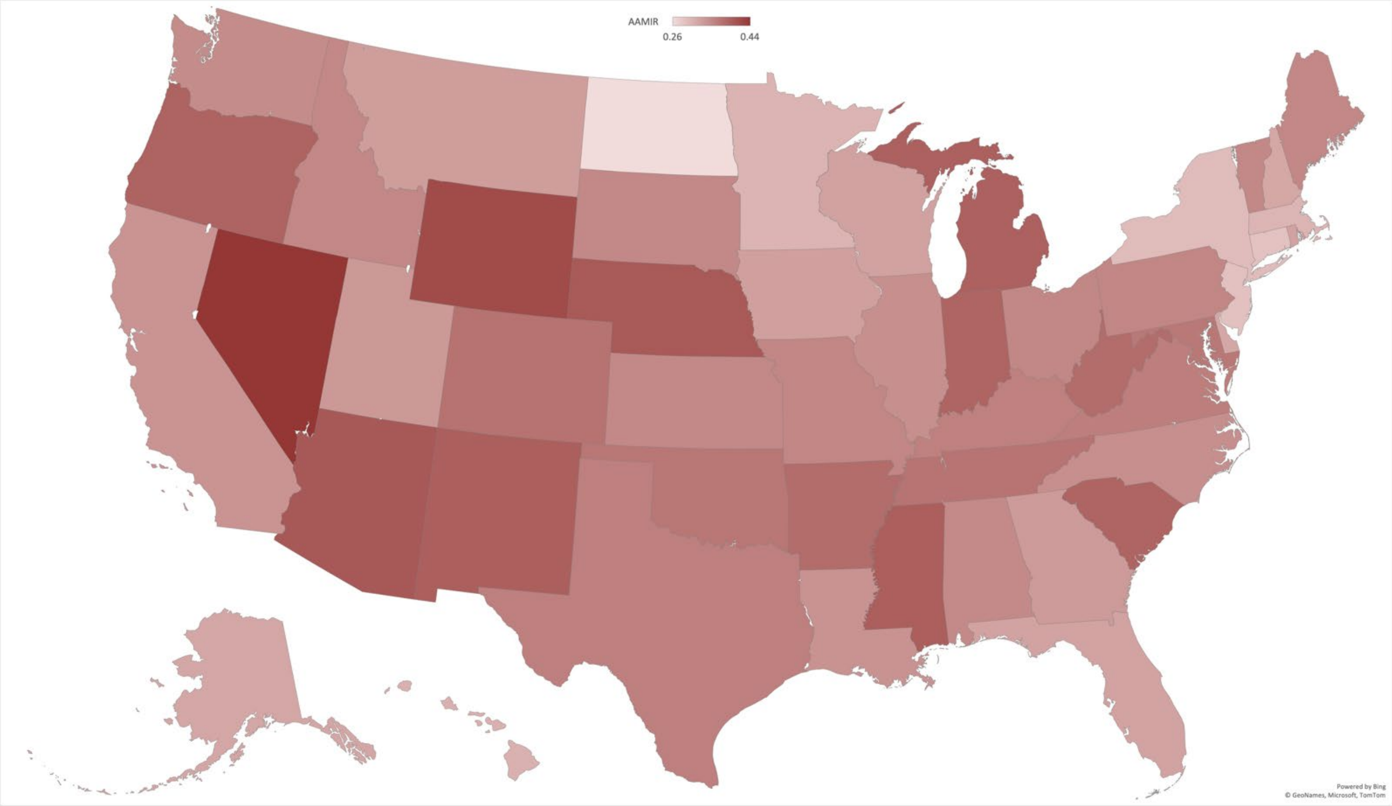

Supplement: Supplementary file 1 [file diseases-13-00392-s001.zip › diseases-3950574-supplementary.pdf]
