# Supplementary figures and images for: Colorectal Cancer in the U.S., 1999–2021: Declining Rates, Rising Concerns, and Persistent Disparities
Source: Diseases. 2025 Dec 4;13(12):392. doi: 10.3390/diseases13120392 (PMC12731979; doi:10.3390/diseases13120392)

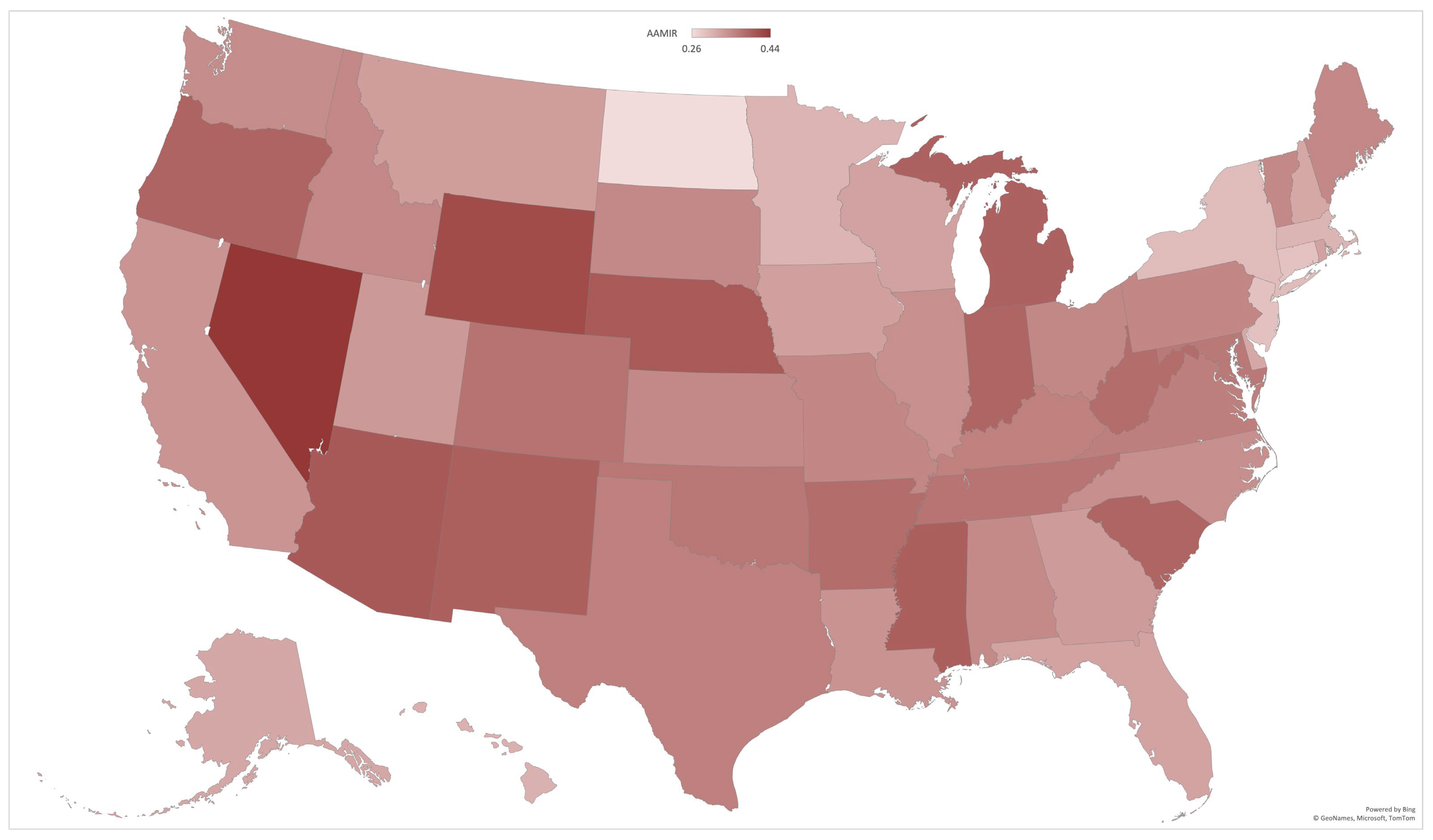

Supplement: Supplementary file 1 [file diseases-13-00392-s001.zip › Supplemental Figure 1.tif]
